# First Record of the Rare Species *Aeromonas lusitana* from Rainbow Trout (*Oncorhynchus mykiss*, Walbaum): Comparative Analysis with the Existing Strains

**DOI:** 10.3390/pathogens11111299

**Published:** 2022-11-05

**Authors:** Ana Fernández-Bravo, Vicente Vega-Sánchez, Alba Pérez-Cataluña, Fadua Latif-Eugenín, Roxana Beaz-Hidalgo, Antonio Martínez-Murcia, Edgardo Soriano-Vargas, Omar Alejandro Cabrero-Martínez, Graciela Castro-Escarpulli, Maria José Figueras

**Affiliations:** 1 Unitat de Microbiologia, Departament de Ciènces Médiques Bàsiques, Facultat de Medicina i Ciències de la Salut, IISPV, Universitat Rovira i Virgili, 43201 Reus, Spain; 2 Instituto de Ciencias Agropecuarias, Universidad Autónoma del Estado de Hidalgo, Tulancingo 43600, Mexico; 3 Área de Microbiología, EPSO, Universidad Miguel Hernández, 03300 Orihuela, Spain; 4 Centro de Investigación y Estudios Avanzados en Salud Animal, Facultad de Medicina Veterinaria y Zootecnia, Universidad Autónoma del Estado de México, Toluca 50295, Mexico; 5 Laboratorio de Investigación Clínica y Ambiental, Escuela Nacional de Ciencias Biológicas (ENCB), Instituto Politécnico Nacional (IPN), Mexico City 11350, Mexico

**Keywords:** *Aeromonas lusitana*, isDDH, ANI, virulence genes, core genomes, citrate synthase

## Abstract

The species *Aeromonas lusitana* was first described in 2016 with five strains recovered from untreated water and vegetables from Portugal. Since then, no further records exist of this species. During a surveillance study on the presence of *Aeromonas* in fish farms in Mexico, a new strain (ESV-351) of the mentioned species isolated from a rainbow trout was recovered. It was identified because it clustered phylogenetically with the type strain of *A. lusitana* based on the analysis of the *rpo*D gene sequences. In the present study, phenotypic characteristics, antimicrobial resistance profiles, and the presence of putative virulence genes of this novel strain (ESV-351) were determined in parallel to the five isolates from the original species description. Phenotypic differential characteristics exhibited by *A. lusitana* ESV-351 depicted an evident similarity to the characteristics exhibited by the other evaluated strains. However, the novel strain was positive for the production of indole using conventional methods, while the rest of the strains, including the type strain, were negative for its production. Furthermore, intermediate resistance to ampicillin, amoxicillin-clavulanic acid and cephalothin was detected in both the novel and the type strain. Five different virulence-related genes were detected in the novel strain and in the previously described strains, with the type strain exhibiting the highest number of virulence-related genes. In addition to this, the genome of the novel strain (ESV-351) was sequenced and compared with the genomes from the type strain (*A. lusitana* CECT 7828^T^) and other *Aeromonas* spp. The genomic analysis defined *Aeromonas tecta* as the closest species to *A. lusitana* with a highly similar number of predicted proteins. The genomic size, the number of protein-encoding genes and the number of different tRNAs, among other characteristics, make it possible to propose that the ESV-351 strain could potentially have the capacity to adapt to different environments. Genome comparison of the ESV-351 strain with the type strain revealed that both possess a similar sequence of the citrate synthase gene. In addition to this finding, the chromosomal region containing the citrate synthase locus of the novel strain exhibits some similarity to the chromosomal region in the genome of the *A. hydrophila* type strain and other known human pathogens, such as *Vibrio cholerae*. This could suggest a possible virulence role for the citrate synthase gene in *A. lusitana* (ESV-351).

## 1. Introduction

The genus *Aeromonas* resides within the *Aeromonadaceae* family which belongs to the *Gammaproteobacteria* class [1]. This genus includes species autochthonous to aquatic habitats and widely distributed in water sources, with some being associated with fish and human diseases [2,3,4,5,6,7,8,9].

Reported virulence genes detected in *Aeromonas* pathogenic strains are known to be acquired from a common ancestor or through horizontal gene transfer (HGT) [10]. However, *Aeromonas* virulence is considered multifactorial and related to different structures implicated in adhesion, such as flagella, fimbria, outer membrane proteins (OMPs), and capsule biosynthesis, a structure that mediates the first contact between the host and pathogen cells and the subsequent evasion of the host immune response [3,11]. Different extracellular secreted proteins, including cytotoxic and cytotonic enterotoxin, hemolysin, proteases, and lipases play an important role in bacterial invasion and infection establishment [3,12,13,14]. In addition to this, four out of six types of secretion systems described in Gram-negative bacteria have been detected in the *Aeromonas* genus (type II, III, IV and VI). Type III (T3SS) is considered to be of high importance in the pathogenesis and virulence of disease-generating *Aeromonas* strains isolated from fish and humans [2,15].

The taxonomy of the *Aeromonas* genus has rapidly evolved in recent years with the addition of new species. Currently, the genus includes 32 species, and 4 are pending to be described [15]. *Aeromonas lusitana* was first described in 2016, based on the analysis of five isolates recovered from untreated water and vegetables from Portugal [16]. In order to increase the knowledge about this recently discovered species, the draft genome sequence of *A. lusitana* CECT 7828^T^ type strain was recently sequenced and deposited in the DDBJ/ENA/GenBank under the accession number PGCP00000000 [17].

In a previous surveillance study that investigated the presence of *Aeromonas* in fish farms in Mexico, a strain (ESV-351) was isolated from the gills of a rainbow trout that phylogenetically clustered with *A. lusitana* [18]. In the present study, we analyzed the characteristics of the identified ESV-351 new strain in parallel with the five already known strains of this species, comparing biochemical features, antimicrobial activity, and the presence of putative virulence genes. In addition to this characterization, we compared the genomic sequence of this novel strain with the already available draft sequence of the type strain. The main aim of this study is to obtain a better knowledge of this rarely isolated species.

## 2. Materials and Methods

### 2.1. Bacterial Strains

A juvenile cultured rainbow trout collected from a pond located in “Huasca de Ocampo” in the state of Hidalgo in Mexico was examined for bacterial presence in a previous study [19]. Samples from gills, intestine, spleen, liver and kidney were plated on Tryptic Soy Agar (TSA, Difco^TM^, Le Pont de Claix, France) growth medium and incubated at 30 °C for 24 h. Colonies were confirmed using the protocols described by Vega-Sánchez et al. [18], and a phylogenetic analysis based on the *gyrB* and *rpoD* gene sequences of the obtained strains, revealed that one strain (ESV-351) from gills clustered with the type strain of *A. lusitana* recovered from water (A.11/6T = DMSZ 24095T = CECT 7828^T^; GenBank accession number *gyrB*: KJ743554; *rpoD*: KJ743565).

For the majority of the analysis, the ESV-351 strain was compared with the five strains and for the studies of the phenotypic characteristics and presence of virulence genes, the four additional strains included in the initial description of *A. lusitana*, recovered from untreated water and vegetables in Portugal [16] were used. Three strains (A.11/6T, A.136/15, and A.28/6) were isolated from untreated water and the other two (L8-3 and L10-4) were isolated from vegetables intended for human consumption [20].

### 2.2. Phenotypic Characterization and Antimicrobial Susceptibility Testing

Phenotypic tests of the Mexican strain ESV-351 were performed by conventional biochemical tests, as reported in previous studies such as oxidase, indole, citrate utilization, or growth in NaCl. Appropriate positive and negative controls were included for each test and with each lot of prepared medium [21,22]. Subsequently, our strain was compared with the strains employed for the definition of *A. lusitana* [16]. Additionally, we performed a comparison between the results obtained using conventional biochemical tests (i.e., individual separated tests) and through the automatized MicroScan WalkAway-40 system using the ESV-351 strain. Furthermore, antimicrobial susceptibility testing of 23 antibiotics was evaluated with the MicroScan WalkAway-40 system and with the Kirby–Bauer disc diffusion method. Results were interpreted according to the Clinical Laboratory Standards Institute (CLSI) guidelines [23]. Both the phenotypic tests and antimicrobial susceptibility tests were performed in triplicate.

### 2.3. Fingerprinting by ERIC-PCR

An enterobacterial repetitive intergenic consensus PCR (ERIC-PCR) was performed with the primers and conditions previously described by Versalovic et al. [24]. Gel images were saved as TIFF files and further analyzed wit BioNumerics software, version 1.5 [16].

### 2.4. Putative Virulence Genes Detection

The Mexican strain ESV-351 and the five strains described by Martínez-Murcia et al. [16] were evaluated by PCR for the presence of nine putative virulence genes. The surveyed genes were: *laf* which encodes for the lateral flagellum biosynthesis [25]; *aer*A encoding for the production of aerolysin/hemolysin [26]; *act* encoding for the production of the cytotoxic enterotoxin [27]; *ast* which encodes the synthesis of the heat-stable enterotoxin [28]; *alt* involved in the production of the heat-labile cytotonic enterotoxin [29]; the lipase-encoding genes *lip*, *lip*H3, *pla* and *plc* [30]; the serine protease encoding genes *asp*A and *ahe*2 [31]; the *ascF*-*ascG* and *ascV* genes which encode components of a putative T3SS in *Aeromonas* [32]; the effector protein *aexT* which encodes for the ADP-ribosyltransferase toxin [33] and *aopP*, which encodes for the AopP protein that usurps the innate immune signaling of the host [34]; and lastly the Shiga toxin encoding genes *stx1* [35] and *stx2* [36].

### 2.5. Genome Sequencing, Assembling and Annotation

Genomic DNA extraction of the Mexican strain ESV-351 was performed using the Easy-DNA^TM^ gDNA purification kit (Invitrogen, Madrid, Spain). DNA integrity of the extracted genomic material was confirmed on 1.5% agarose gel electrophoresis and the DNA was quantified with the Qubit^TM^ Fluorometer using the Qubit^®^ Broad-Range Assay kit (Invitrogen). Genomic libraries for paired-end sequencing were performed with the Nextera^®^ DNA Library Preparation kit (Illumina, Lisbon, Portugal) and sequenced with the Illumina MiSeq System. Clean reads were assembled de novo with the CGE Assembler 1.2 which uses the Velvet algorithm package for Illumina sequence data [37]. Contig annotation was performed with the Rapid Annotation Subsystem Technology (RAST) server (http://rast.nmpdr.org, accessed on 25 April 2019) [38,39].

### 2.6. Phylogenetic Analysis Based on the Genome

The phylogenetic analysis was performed to confirm the clustering of the ESV-351 strain within the *A. lusitana* CECT 7828^T^ type strain and to analyze phylogenetic relation to other species. A total of 7 housekeeping genes (*rpoD, gyrB, recA, gyrA, atpD, dnaJ* and *dnaX*) were extracted from the genome of the ESV-351 strain using the Basic Local Alignment Search Tool from the National Center for Biotechnology Information (NCBI), to perform a Multilocus Phylogenetic Analysis (MLPA). Alignment of the concatenated sequences of these genes was performed using the ClustalW and the phylogenetic tree was constructed by the Maximum-likelihood method with MEGA software, V6.0.

### 2.7. Genome Information and Comparative Analysis

The genome-derived curated data of *A. lusitana* ESV-351 (GenBank accession in process) was collected from the SEED viewer from RAST (http://rast.nmpdr.org/seedviewer.cgi, accessed on 15 May 2019) [38,39] and from the tRNAscan-SE web server for the search of tRNA genes in genomic sequences (http://lowelab.ucsc.edu/tRNAscan-SE/, accessed on 15 May 2019) [40]. Additional genomes were employed for the comparative analysis of the obtained information, such as the genome of *A. lusitana* CECT 7828^T^ type strain (GenBank Whole-Genome Shotgun project no. PRJNA417247) isolated from untreated water [16], as well as genomes of the phylogenetically closest species: *A. eucrenophila* CECT 4224^T^ (GenBank Whole-Genome Shotgun project no. CDDF00000000.1) isolated from freshwater fish [41]; *A. tecta* CECT 7082^T^ (GenBank Whole-Genome Shotgun project no. CDCA00000000.1) isolated from children feces; *A. aquatica* CECT 8025^T^ (GenBank Whole-Genome Shotgun project no. JRGL00000000.1) isolated from Finnish waters associated to cyanobacterial blooms [42] and lastly, *A. encheleia* CECT 4342^T^ (GenBank Whole-Genome Shotgun project no. CDDI00000000.1) isolated from European eels [43]. These genomes were annotated in the SEED viewer from RAST to obtain the curated annotation data, while tRNAscan-SE was employed to obtain predicted tRNA genes. Moreover, previously described complete genomes were also taken into consideration, such as *A. hydrophila* ATCC 7966^T^ (GenBank accession no. CP000462.1), isolated from canned milk with a fishy odor [44]; *A. salmonicida* A449 (GenBank no. CP000644.1), isolated from a brown trout with furunculosis [45]; *A. salmonicida* 01-B526 (GenBank Whole-Genome Shotgun project no. AGVO00000000.1) isolated from infected brook trout in Canada [46]; the formerly called *A. aquariorum* AAK1 (GenBank Whole-Genome Shotgun project no. BAFL00000000.1), currently reclassified as *A. dhakensis*, which was isolated from the blood of a patient with septicemia and necrotizing fasciitis (NF) [47]; *A. veronii* B565 (GenBank accession no. CP002607.1), isolated from an aquaculture pond sediment in China [48]; and *A. caviae* Ae398 (GenBank Whole-Genome Shotgun project no. CACP00000000.1), isolated from the feces of a child with profuse diarrhea [49].

### 2.8. Genomic Indexes

The genomic sequences of *A. lusitana* ESV-351 and the type strain CECT 7828^T^ were uploaded to the Genome-to-Genome Distance Calculator GGDC 2.0 web server (http://ggdc.dsmz.de/distcalc2.php, accessed on 5 December 2019), to perform an *in silico* DNA-DNA hybridization (*is*DDH) [50]. Additionally, the Average Nucleotide Identity (ANI) values between *A. lusitana* ESV-351, *A. lusitana* CECT 7828^T^, and the closest species were calculated through the OrthoANI algorithm and software [51].

### 2.9. Pangenome and Core Genes Analysis

The pangenome represents the entire set of the total genes found across all isolates compared from a given species and can be subdivided into the genes shared by all of them (core genome) and the genes present only in some members of a species (accessory genes) [52]. Analysis of specific pangenomes could reveal novel genes involved in specific functions, such as pathogenicity-related genes, stress response and antimicrobial resistance, among other functions [53]. In order to estimate the number of genes present in the pangenome and the core genome, we employed the previously described pan-core plot method [53,54]. The core-pan genome was calculated employing the genome annotations performed with the gene prediction software Prodigal [55] through its input to the BLAST-dependent program *pancoreplot* from CMG Biotools [56].

Gene families with at least one gene in common in the genomes compared were plotted in the core genome. The cut-off value was previously established at 50/50%, indicating that two genes belong to the same gene family if their amino acids are 50% identical over 50% of the length of the longest gene (50% identity/50% coverage). The rest of the total genes not included in the core genome were plotted in the pangenome [56,57].

### 2.10. Comparison of Predicted Protein Sequences

Assembled genomes in FASTA format annotated in the RAST server were compared on the SEED viewer [38,39]. The SEED viewer was used to compare the sequence identity of the annotated predicted proteins of the new *A. lusitana* strain ESV-351 and the closest species based on the sequence of the *A. lusitana* type strain (CECT 7828^T^), using the “sequence-based comparison” from the available comparative tools.

### 2.11. Protein Count and Distribution in Subsystems

A search of associated proteins to different functional and structural pathways was performed with the genomes of the ESV-351 strain and the *A. lusitana* CECT 7828^T^ type strain, in addition to the *A. tecta* CECT 7082^T^ type strain, the closest known species. These proteins were evaluated after their annotation in the RAST server [38,39].

### 2.12. Citrate Synthase Sequence Analysis in A. lusitana

The gene encoding for the citrate synthase protein was searched in the genomes of *A. lusitana* ESV-351 and CECT 7828^T^ through the RAST server with the SEED viewer. Based on the nucleotide sequence obtained from this gene in the *A. lusitana* type strain (CECT 7828^T^), we performed a search in all the available genomes of the *Aeromonas* genus, using the BLAST algorithm. By this means, a phylogenetic tree was constructed with the protein sequences obtained from the performed search using the Neighbor-Joining clustering method. In order to evaluate the possible function of this protein as a virulence factor in *A. lusitana,* the tool “function-based sequence” from the SEED viewer was used to compare the locus of the citrate synthase gene in *A. lusitana* ESV-351 and the region surrounding this gene with similar organisms in the SEED viewer database.

## 3. Results and Discussion

### 3.1. Fingerprinting by ERIC-PCR

**Figure 1:**
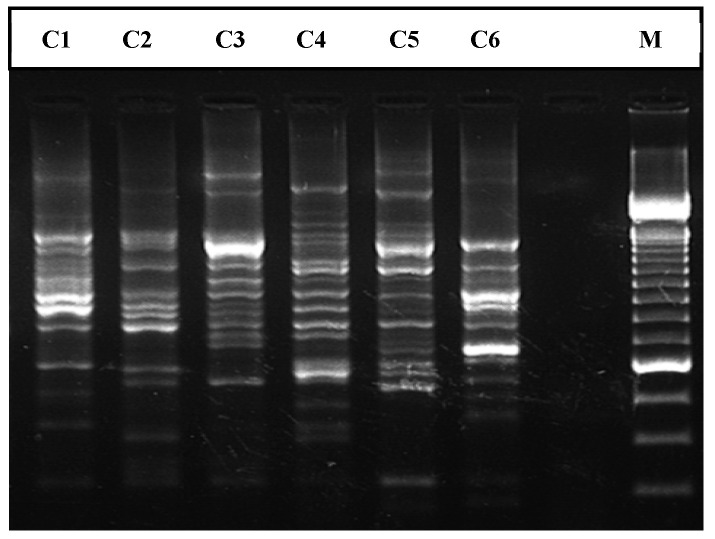
ERIC-PCR profile of the six *Aeromonas lusitana* strains included in the present study. C1: 11/6^T^ (=MDC 2473^T^ = DSM 24095^T^ = CECT 7828^T^); C2: A.136/15 (MDC 2467); C3: L8-3 (MDC 2468); C4: L10-4 (MDC 2469); C5: A.28/6 (MDC 2472); C6: ESV-351; M: Molecular weight. C1, C2 and C5 were recovered from water; C3 and C4 were isolated from vegetables. C6, was recovered from rainbow trout.

The comparison of the ERIC-PCR fingerprint profiles obtained with the discovered strain (ESV-351) from rainbow trout and the strains from water and vegetables included in the description of *A. lusitana*, revealed very distinctive patterns indicating a great genetic diversity (Figure 1).

### 3.2. Phenotypic Characterization and Antimicrobial Susceptibility Testing

All phenotypic characteristics obtained by conventional biochemical testing and the MicroScan WalkAway-40 method of the novel ESV-351 strain and selected strains of *A. lusitana* are shown in Table 1 and Supplementary Table S1. The only discordant phenotypic characteristic of the ESV-351 strain in relation to the other *A. lusitana* strains was the positive result for indole production (Table 1 and Supplementary Table S1). In fact, the four selected strains and the *A. lusitana* (CECT 7828^T^) type strain exhibited negative results for indole production as described by Martínez-Murcia et al. [16] when evaluated with a conventional method. However, the indole production showed to be positive with the MicroScan WalkAway-40 identification system. Therefore, this should be taken into consideration when trying to identify *Aeromonas* strains, since the negative indole production was considered a key test for the phenotypic differentiation of *A. lusitana* from other species [16]. Other discordant results were observed between conventional biochemical tests and MicroScan WalkAway-40 systems, such as the ADH test, LDC, ONPG, and citrate utilization (Supplementary Table S1). Previous studies have indicated that the MicroScan WalkAway system tends to confuse the *Aeromonas* genus with other genera, such as *Pasteurella, Pseudomonas,* and *Vibrio* [58].

The susceptibility testing of the 23 antimicrobial agents evaluated exhibited intermediate resistance to ampicillin, amoxicillin-clavulanic acid, and cephalothin (Table 2). These results agree with the classical resistance to ampicillin defined as a typical characteristic in the majority of the species in the *Aeromonas* genus [21,59,60]. A comparative study of antimicrobial susceptibility of *Aeromonas* spp. isolates obtained from environmental and clinical samples by Aravena-Román et al. [61] described that 83% (161/193) of the strains were resistant to amoxicillin-clavulanic acid; 94% (135/144) of clinical strains and 53% (26/49) of environmental strains. In addition, 73% (140/193) of the strains also exhibited resistance to cephalothin; 79% (114/144) of the clinical strains and 53% (26/49) of the environmental strains.

### 3.3. Putative Virulence Genes Detection

The six strains of the novel *A. lusitana* species, exhibited five different virulence gene profiles (Table 3) with the CECT 7828^T^ type strain harboring the higher number of virulence-related genes (*laf* ^+^/*lipase* ^+^/*serine* ^+^/*ascF-ascG* ^+^/*ascV* ^+^/*aexT* ^+^). Lye [62] demonstrated that *Aeromonas* strains isolated from environmental samples were pathogenic and harbored virulence abilities similar to those observed in clinical isolates on a murine model.

The lipase-related genes (*lip*, *lip*H3, *pla,* and *plc*) were detected in all the surveyed strains (Table 3). These enzymes have a hydrolytic effect on the membrane lipids of the host cells, and lipase-related genes have been shown to be highly detected in clinical (93%) and environmental isolates (96%) [30]. In fact, Merino et al. [63] reported that insertion mutants of *A. hydrophila* AH-3 defective in either *pla* or *plc* genes were defective in phospholipase A1 and C activities, respectively, effectively reducing its lethal dose in murine and fish models. The aerolysin-encoding gene *aerA*, was present in all strains. This gene has been reported in 96% (74/77) of *Aeromonas* spp. isolates from fish [31], ranging from 76% to 81% in clinical isolates and 68% to 78% in environmental isolates [30,64]. The serine protease genes were detected in 83% (5/6) of the strains (Table 3). These genes play an important role in *Aeromonas* pathogenicity since these proteins mediate tissue damage and facilitate bacterial invasion into the host cell [3]. Although, it has been reported that isolates of *A. taiwanensis* and *A. sanarelii* from chironomid egg masses exhibited a low or no presence of this gene (25%) [59]. The presence of the lateral flagellum encoding gene (*laf*) detected in our study was 67% (Table 3), this is an important structure involved in adherence and biofilm formation, expressed during bacterial growth on viscous surfaces [65]. However, in a previous study, the *laf* gene was detected in 100% of the *Aeromonas* spp. isolates from drinking water in Spain [66]. Aravena-Román et al. [64] observed that the presence of the *laf* gene was more prevalent in clinical isolates than in environmental ones. Furthermore, the cytotoxic enterotoxin encoding gene (*act*) was detected in 50% of the six studied strains, this percentage is similar to those reported in previous studies, this is, 43% to 62% of the strains harboring the gene [67,68,69]. On the other hand, the genes that encode components of the T3SS of *Aeromonas* (*ascF*-*ascG* and *ascV*) were detected in three of the six strains (CECT 7828^T^, MDC 2467, MDC 2468). However, simultaneous detection of the encoding gene for the effector T3SS protein *aexT* was present only in the type strain (Table 3). Nevertheless, none of the six strains bared the gene encoding the *aop-P* toxin, also delivered by the T3SS. The T3SS is considered an important virulence factor since it injects effector toxins directly into the cytoplasm of host cells [3]. Previous studies in which different T3SS effector genes were inactivated in mutant *A. salmonicida* subsp. *salmonicida* strains demonstrated that the T3SS is essential for its toxicity and virulence [70,71]. In addition, the mutant strains showed to be less virulent [71]. In none of the six strains were the genes encoding for the heat-stable enterotoxin (*ast*), the heat-labile cytotonic enterotoxin (*alt*), or the Shiga toxins (*stx1* and *stx2*) detected (Table 3).

### 3.4. Phylogenetic Analysis with 7 Housekeeping Genes Extracted from the Genomes

**Figure 2:**
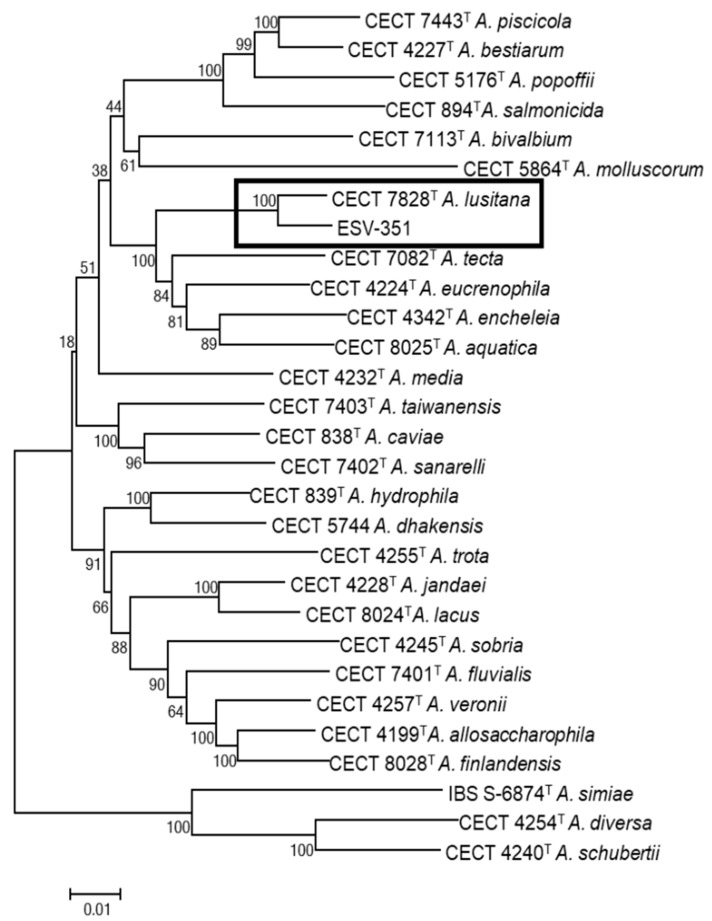
Phylogenetic tree derived from the MLPA of the concatenated sequences of 7 housekeeping genes (3849 bp) from the *Aeromonas* genomes (*gyrB*, *rpoD*, *recA*, *dnaJ*, *gyrA*, *dnaX* and *atpD*). Notice the cluster formed by the type strain of *Aeromonas lusitana* CECT 7828^T^ and *A. lusitana* ESV-351 in relation to the type strains of the other *Aeromonas* species. Numbers at nodes indicate bootstrap values (percentage of 1000 replicates). Bar 0.01 estimated nucleotide substitutions per site.

The use of phylogenetic analysis based on concatenated housekeeping genes has been extensively recommended for accurate identification of *Aeromonas* species [16]. The neighbor-joining phylogenetic tree constructed with the complete sequence concatenation of seven housekeeping genes (*gyrB*, *rpoD*, *recA*, *dnaJ*, *gyrA*, *dnaX,* and *atpD*) extracted from the ESV-351 genome and from the genomes of the *Aeromonas* type strains, ratified that the ESV-351 strain belongs to the *A. lusitana* species (Figure 2). This confirmed the results obtained from the concatenation of the *rpoD* and *gyrB* genes in a previous study [18]. The MLPA analysis showed that *A. lusitana* formed a clade with the species *A*. *encheleia* CECT 4342^T^, *A. eucrenophila* CECT 4224^T^, *A. aquatica* CECT 8025^T^, and *A. tecta* CECT 7082^T^ (Figure 2). Apart from the species *A. aquatica* (CECT 8025^T^) that was described after *A. lusitana*, the other three were previously reported as the closest species to *A. lusitana* [16,18]. Genomes of these closely related species were further evaluated with comparative tools.

### 3.5. Comparison of ANI and isDDH Values Obtained between A. lusitana and Its Closest Species

**Figure 3:**
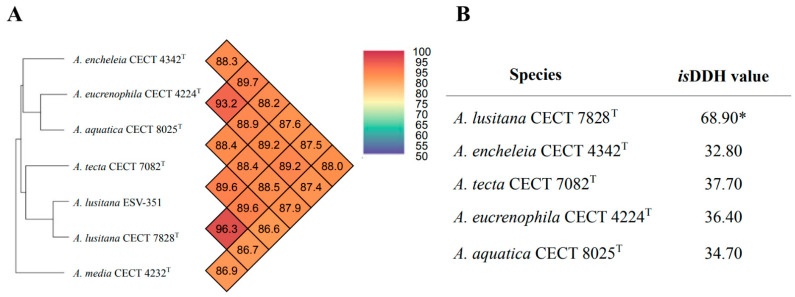
Comparative results of the Average Nucleotide Identity obtained between the genomes of the *Aeromonas lusitana* strain, isolated from gills of rainbow trout from *Mexico* (ESV-351), the type strain recovered from untreated water from Portugal (CECT 7828^T^), the closest *Aeromonas* species on the based on the MLPA (Figure 2) and *Aeromonas media* a relatively distant species (**A**). Results of the *is*DDH results between the genome of the strain ESV-351, the type strain (CECT 7828^T^) and the closest *Aeromonas* species. * Belongs to the same species (**B**).

Generally, for the definition of different species, an ANI below 96% [51] and an *is*DDH value below 70% [50] are considered. The ANI and *is*DDH values between *A. lusitana* ESV-351 and its closest species were below 90 and 40%, respectively (Figure 3), similar to previous results reported among *Aeromonas* species [4,41]. As shown in Figure 3A, the ANI value obtained between the ESV-351 and the *A. lusitana* type strain genomes was higher than 96% (96.3%), and the *is*DDH values between these strains were less than 70% (68.9%) (Figure 3B). An explanation of this borderline result could be that these strains are the same species, but with genomic differences between them. Colston et al. [41], obtained similar *is*DDH results with strains belonging to *A. allosaccharophila* species, describing the taxonomy of this species as controversial. For this reason, it is possible that the *A. lusitana* species could be a controversial species such as *A. allosaccharophila*. Therefore, it is important to obtain additional genomic sequences from *A. lusitana* strains to determine the particular *is*DDH cut-off value for this novel species (68.9% = 69%). However, ANI results are more reliable, and it has been proposed as a new standard to define microbial species, gaining wide acceptance as an essential parameter [41,72].

### 3.6. Genomic Information and Its Comparison to Other Related and Non-Related Species

**Figure 4:**
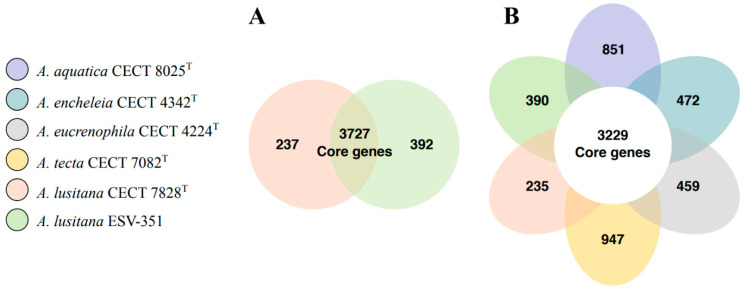
Pangenome and core genome comparison between the *A. lusitana* (ESV-351) strain isolated from gills of a rainbow trout from Mexico and the type strain of the species recovered from water in Portugal (CECT 7828^T^) (**A**) and its closest related *Aeromonas* species (**B**). The clockwise order goes from the most distant to the most genetically closely related species to *A. lusitana* based on the MLPA (Figure 2).

Figure 4 shows a genomic comparison of *A. lusitana* ESV-351 and type strain CECT 7828^T^ with the published genomes of its closest species, i.e., *A. tecta*, *A. eucrenophila, A. encheleia, A. aquatica,* and of six additional *Aeromonas* species [44,45,46,47,48,49] is shown. As shown in this figure, the ESV-351 strain exhibited a slightly larger genome size (4.74 Mb) than the type strain (4.55 Mb), and similar to its closest species *A. tecta* (4.75 Mb) to several other species (Table 4). Moreover, the G+C content of the ESV-351 strain (60.7%) was within the range (mol% G+C 57 to 63) that has been reported for the *Aeromonas* genus [3]. The number of predicted tRNAs in ESV-351 strain was 110, the same number present in the complete genome of *A. salmonicida* A449 [45], and higher than the 98 detected in the genome of *A. lusitana* CECT 7828^T^ type strain and its closest species, which ranges between 82 to 101. Pang et al. [73] performed comparative genomic analyses of five epidemic strains of *A. hydrophila* belonging to sequence type (ST) 251 with *A. hydrophila* ATCC 7966^T^ and reported that the latter possesses a higher number of tRNAs than the other strains. These authors give support to a previous report indicating that a higher number of tRNAs may be useful to increase the protein synthesis [73,74]. Considering the latter arguments, *A. lusitana* ESV-351 could also synthesize a higher number of tRNAs than *A. lusitana* CECT 7828^T^ or its closest species. 

In relation to the protein-coding genes, *A. lusitana* ESV-351 exhibited a higher number (4251) than the CECT 7828^T^ type strain (4069) (Table 4). The first described genomes of two species considered as important pathogens to fish and humans, *A. salmonicida* A449 and *A. hydrophila* ATCC 7966^T^, exhibited a higher number of protein-coding genes, 4388 and 4128, respectively [44,45]. The higher number of genes encoded by *A. salmonicida* A449, were related to the potential virulence of this species, with an abundance of insertions and pseudogenes, which could suggest an adaptative evolution of this strain to different hosts [45]. In the case of *A. hydrophila* ATCC 7966^T^, some genes encode several metabolic pathways that allow the strain to survive in different environments [44]. This information allows us to consider that *A. lusitana* could adapt to different environments, due to its high number of protein-coding genes enabling the species to express a greater number of genes related to cell division virulence, or metabolism. It is noteworthy that *A. lusitana* ESV-351 possesses a higher number of these proteins than the type strain *A. lusitana* CECT 7828^T^ (Table 4). An explanation for this could be that the higher number of protein-coding genes could be related to the origin of the strain, in an evolutionary and adaptative manner to its environment. The ESV-351 strain [18] was isolated from a rainbow trout in Mexico, while the CECT 7828^T^ [16] was isolated from untreated water in Portugal. A significant difference in stress-inducing factors could define the need from the bacterium to adapt to its host. Considering these results and comparing them with the similar characteristics found in *A. salmonicida* A499 and *A. hydrophila* ATCC 7966^T^, the strain *A. lusitana* ESV-351 could also be suggested to be a potential important fish pathogen, despite the fact that the strain was recovered from apparently normal gills. Gills are considered an important colonization site in rainbow trout and other fish during the infective process [75]. Therefore, more studies are needed to demonstrate the capacity of *A. lusitana* ESV-351 to infect fish and to determine if the encoded proteins correspond to virulence genes, or to different metabolic pathways that could increase the pathogenicity of the strain in fish.

### 3.7. Pangenome and Accessory Genome Analysis

The pangenome and core genes of the surveyed *A. lusitana* ESV-351 and CECT 7828^T^ strains were obtained and later determined with the closest species (*A. aquatica* CECT 8025^T^, *A. eucrenophila* CECT 4224^T^, *A. encheleia* CECT 4342^T^ and *A. tecta* CECT 7082^T^). Both were calculated and analyzed to further obtain data about the novel *A. lusitana* ESV-351 strain. We identified a pangenome of 4356 genes and a core genome of 3727 genes between the *A. lusitana* ESV-351 and CECT 7828^T^ genomes (Figure 4A). Taking into consideration the average gene numbers of these two strains (4160), the 3727 core genes represent approximately 89% of the total genome. In other words, an elevated number of genomic regions between both strains is conserved. In addition to this, *A. lusitana* ESV-351 presented a higher number of unique accessory genes (strain-specific) than the type strain *A. lusitana* CECT 7828^T^, 392 and 237 genes, respectively. 

Next, we identified the pangenome of the six surveyed genomes comprising 6583 genes and a core genome of 3229 core genes. The average gene number was 4138 for the six strains, the 3229 core genes represent approximately 78% of the genome, indicative of highly conserved genomic features between the analyzed genomes. As shown in Figure 4B, *A. tecta* CECT 7082^T^ [76] exhibited a higher number of unique genes (947), this could indicate that some of these accessory genes are related to its survival and adaptation to detrimental environments, as previously reported [77]. This consideration is established from the premise that the strain was isolated from the feces of a child with diarrhea [76], exposing the strain to stress induced by the immune response elicited by the host. Further pathogenicity tests and data analysis should be performed, to elucidate if these genes encode putative virulence factors contributing to its survival.

### 3.8. Comparison of Predicted Protein Sequences

**Figure 5:**
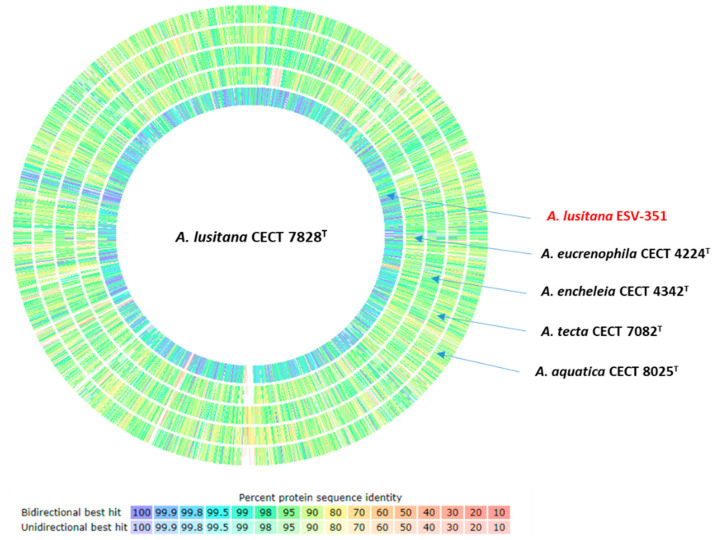
Comparison of predicted proteins. Protein sequences were compared by using the Rapid Annotation System Technology (RAST)-SEED viewer sequenced-based comparison tool with *Aeromonas lusitana* CECT 7828^T^ used as the reference sequence. Protein sequence identity (%) is expressed by color coding provided in the legend. A threshold of 98% sequence identity was used to identify highly conserved sequences.

Protein sequences of all predicted open-reading frames (ORFs) detected in the genome of *A. lusitana* CECT 7828^T^ were used as a reference to compare the predicted protein sequences of *A. lusitana* ESV-351, *A. aquatica* CECT 8025^T^, *A. eucrenophila* CECT 4224^T^, *A. encheleia* CECT 4342^T^, and *A. tecta* CECT 7082^T^ (Figure 5). As expected, strains of *A. lusitana* CECT 7828^T^ and *A. lusitana* ESV-351 were highly similar, with approximately 2538 proteins sharing more than 98% of similarity. Comparison of the *A. lusitana* CECT 7828^T^ type strain with *A. tecta* CECT 7082^T^, its closest species, showed 629 proteins with higher similarity than 98%. In the other three species, the number of proteins with higher similarity than 98% in comparison with *A. lusitana* CECT 7828^T^ was 531 compared with *A. eucrenophila* CECT 4224^T^, 444 with *A. aquatica* CECT 8025^T^ and 304 with *A. encheleia* CECT 4342^T^.

### 3.9. Protein Count and Distribution in Subsystems

**Figure 6:**
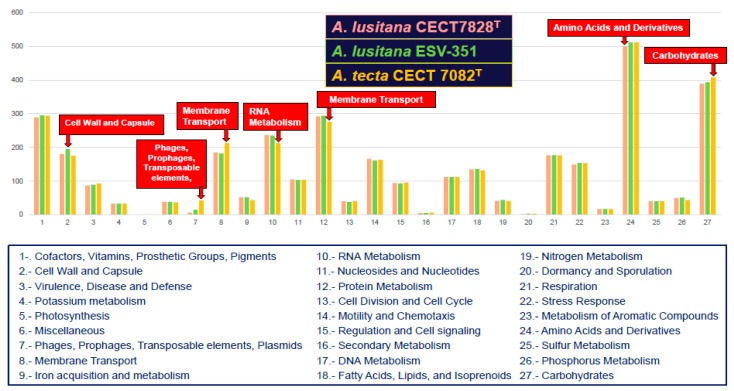
Subsystem category distribution of major protein coding genes of the *Aeromonas lusitana* strains isolated from gills of rainbow trout from Mexico (ESV-351), the type strain recovered from untreated water from Portugal (CECT 7828^T^) and the closest species *Aeromonas tecta* CECT 7082^T^ as annotated by Rapid Annotation System Technology (RAST) server.

A simple comparison between the genome of the *A. lusitana* ESV-351 and CECT 7828^T^ strains and *A. tecta* CECT 7082^T^ was performed based on subsystems. Subsystems represent a group of functional roles, including metabolic pathways, multi-subunit complexes or a specific class of proteins. A total of 4251 protein-coding genes were distributed in 523 subsystems in strain ESV-351, while in strain CECT 7828^T^ the 4069 protein-coding genes were distributed in 521 subsystems. In *A. tecta* CECT 7082^T^, a total of the 4267 protein-coding genes were classified in 525 subsystems. As shown in Figure 6, the three strains (ESV-351, CECT 7828^T^ and CECT 7082^T^) showed a similar distribution associated with different functional or structural systems, such as the relation between the cell wall and capsule, membrane transport, amino acids and their derivatives, and carbohydrates, among the subsystems. The two *A. lusitana* strains showed almost the same number of proteins belonging to different subsystems and as expected, more similar than the number of proteins from its closest species *A. tecta* CECT 7082^T^ (Figure 6). Predicted catabolic pathways of the *A. lusitana* ESV-351 strain include the use of mannose, deoxyribose, ketogluconates, fructose, D-glycerate, and D-ribose as carbon sources, with proteins required for the use of glycerol-3-phosphate, mannitol, and citrate detected as well. The latter could be responsible for the growth capacity in presence of citrate as a carbon source found in the species, which is a described differential phenotypic characteristic [16]. Considering this observation, we decided to screen all the genomes for the presence of the citrate synthase gene.

### 3.10. Citrate Synthase Sequence Analysis in A. lusitana

**Figure 7:**
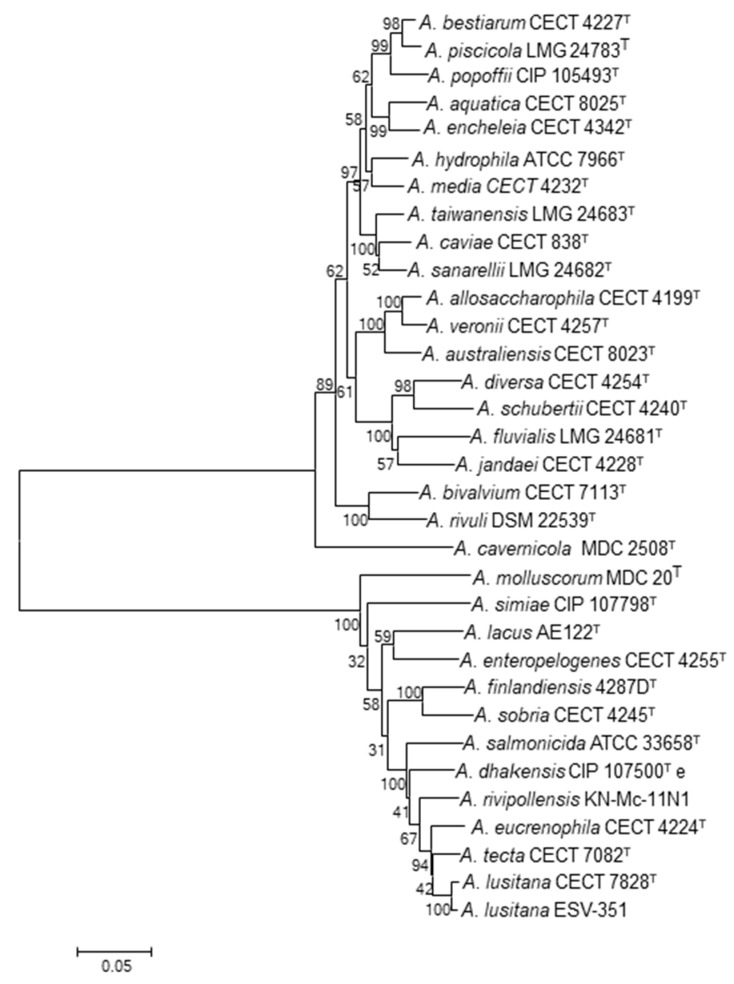
Phylogenetic tree derived from the citrate synthase gene, showing the clusters containing the *Aeromonas lusitana* strain (ESV-351) isolated from Mexico) and the type strain (CECT 7828^T^).

**Figure 8:**
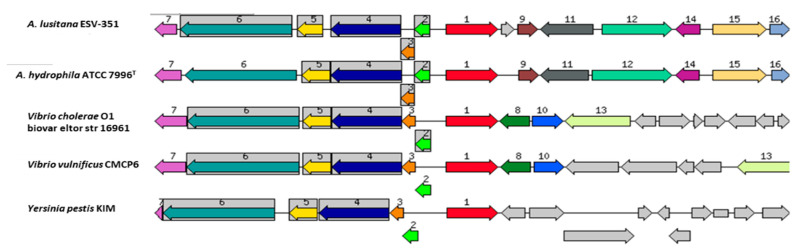
Comparison of the chromosomal region surrounding the conserved citrate synthase locus of *A. lusitana* Mexican strain ESV-351 with the four most similar loci from different bacteria included in the database of the SEED viewer. Orthologous genes are grouped with the same number and color. The number one in red is the citrate synthase gene. Genes whose relative position is conserved in at least four other species are functionally coupled and share gray background boxes. *Aeromonas hydrophila* is the only species of *Aeromonas* that exists at this moment in the database.

The citrate synthase gene was found in all the analyzed genomes of the *Aeromonas* genus, although this gene is not necessarily associated with the use of citrate as a phenotypic characteristic. This is notorious since Martínez-Murcia et al. [16] demonstrated that the phenotypic test for citrate was negative in *A. eucrenophila,* nonetheless, in our study the citrate synthase was found in its genome. In the case of *A. lusitana* ESV-351, the phenotypic character was positive, and the presence of the citrate synthase gene was detected in its genome. Conservation of this gene among all the 32 defined *Aeromonas* spp. was analyzed through the phylogenetic analysis of its sequence extracted from the 32 species, resulting in the formation of two main clusters which group 12 and 20 of the species, respectively, with no apparent pattern observed related to the phenotypic test. Moreover, the phylogenetic analysis showed a similarity of 85 to 97% between species (Figure 7). Additionally, as shown in Figure 8, the citrate synthase encoding gene from *A. lusitana* ESV-351 was compared to the sequences of the latter gene from four important pathogens obtained from the SEED database, resulting in five similar sequences. The analyzed pathogens included the type strain of *A. hydrophila* (ATCC 7966^T^), which is considered an important pathogen for humans and fish [4,15]; *Vibrio cholerae* O1 biovar El tor strain N16961, which was the dominant strain in the seventh global cholera pandemic [78]; *Vibrio vulnificus* CMCP16 strain, responsible for fatal septicemia and necrotizing wound infections [79]; and *Yersinia pestis* KIM, the etiologic agent of bubonic and pneumonic plague [80]. However, as shown in the phylogenetic analysis, the citrate synthase gene found in the type strain was different from the sequence of the ESV-351 strain.

Furthermore, the citrate synthase was described as a virulence factor in other bacteria such as *Agrobacterium tumefaciens* and *Escherichia coli* [81,82]. Additionally, citrate synthase activates a protein that increases survival in *Staphylococcus aureus* [83]. Considering the latter information, it could be possible that the citrate synthase gene could act as an important virulence factor in *A. lusitana* ESV-351, but in contraposition to what could be expected the strain was negative to the citrate utilization test. Final conclusions about the role of this gene should be assessed through in vivo and in vitro pathogenicity tests. In addition, functional analysis of the gene in mutagenic or transcriptional repression conditions could provide further evidence to confirm if, in the strain ESV-351, it can be considered a virulence factor like in other pathogens.

## 4. Conclusions

Here, we demonstrated that phenotypic differential characteristics were highly conserved among the *A. lusitana* strains. However, indole production was different between the novel strain and the type strain. Genomic characterization of *Aeromonas lusitana* ESV-351 exhibited a slightly larger genome size than the type strain, with more protein-coding genes and tRNA, which could facilitate its survival and adaptation to the environment. Furthermore, the chromosomal region surrounding the conserved citrate synthase locus in the novel strain exhibited homology to its counterpart from four pathogenic bacteria, suggesting that this gene could possibly play a role in the virulence of this new strain.

## Figures and Tables

**Table 1:** Phenotypic characteristics ^a^ of the Mexican strain (ESV-351) and the five strains, including the type strain, from the original description of *Aeromonas lusitana* [16].

	ESV-351	CECT 7828^T^	Isolates (4)
β-hemolysis	+	+	+
Indole	+ ^b^	-	-
MR	+	+	+
LDC	+	+	+
Glucose (gas)	+	+	+
Citrate	+	+	+
Hydrolysis of:			
SDS	-	-	V
Aesculin	+	+	+
Starch	+	+	+
Elastase	ND	+	+
Acid from:			
Glycerol	+	+	+
L-arabinose	-	-	-
Salicin	+	+	+
Utilization of:			
DL-lactate	-	-	-
L-arabinose	-	-	-
Growth at: 4.5% NaCl	+	+	+

^a^ Phenotypic characterization was made with conventional methods. ^b^ Negative in two of three repetitions with the MicroScan (Supplementary Table S1). MR: Methyl Red; LCD: Lysine decarboxylase; ND: not done; -: <15% positive; V: between 15 to 85% positive; +: ≥85% of strains positive.

**Table 2:** Antimicrobial susceptibility profiles to 23 antimicrobial agents of the strains ESV-351 and CECT 7828^T^ reported by Martínez-Murcia et al. [16].

Antimicrobial Agent (μg) ^a^	Results (MIC)		Breakpoints		
	ESV-351	CECT 7828^T^	S	I	R
Amikacin (30 μg)	S (≤8)	S (≤8)	≤16	32	≥64
Ampicillin (10 μg)	I (>16)	I (>16)	≤8	16	≥32
Amoxicillin-clavulanic acid (20/10 μg)	I (>16/8)	I (>16/8)	≤8/4	16/8	≥32/16
Aztreonam (30 μg)	S (≤1)	S (≤1)	≤8	16	≥32
Ceftazidime (30 μg)	S (≤1)	S (≤1)	≤8	16	≥32
Ceftriaxone (30 μg)	S (33) ^b^	S (33) ^b^	≥21	14–20	≤13
Cephalothin (30 μg)	I (>16)	I (>16)	≤8	16	≥32
Ciprofloxacin (30 μg)	S (42) ^b^	S (42) ^b^	≥21	16–20	≤15
Cefotaxime (30 μg)	S (≤1)	S (≤1)	≤8	16–32	≥64
Cefoxitin (30 μg)	S (≤8)	S (≤8)	≤8	16	≥32
Ciprofloxacin (5 μg)	S (≤0.5)	S (≤0.5)	≤1	2	≥4
Cefepime (30 μg)	S (≤1)	S (≤1)	≤8	16	≥32
Cefuroxime (30 μg)	S (8) ^b^	S (8) ^b^	≤8	16	≥32
Ertapenem (10 μg)	S (≤0.5)	S (≤0.5)	≤2	4	≥8
Gentamicin (10 μg)	S (≤2)	S (≤2)	≤4	8	≥16
Imipenem (10 μg)	S (≤1)	S (≤1)	≤4	8	≥16
Nalidixic acid (30 μg)	S (≤16)	S (≤16)	≤8	16	≥16
Piperacillin (100 μg)	S (29) ^b^	S (29) ^b^	≥21	18–20	≤17
Piperacillin-tazobactam (100/10 μg)	S (≤8)	S (≤8)	≤16/4	32/4–64/4	≥128/4
Tetracycline (30 μg)	S (27) ^b^	S (27) ^b^	≥19	15–18	≤14
Trimethoprim-sulfamethoxazole (25 μg)	S (≤2/38)	S (≤2/38)	≤2/38	-	≥4/76
Tigecycline (15 μg)	S (≤1)	S (≤1)	≤1	2	≥2
Tobramycin (10 μg)	S (≤2)	S (≤2)	≤2	4	≥4

^a^ Antimicrobial susceptibility tests were performed by the disk diffusion method (results expressed in mm). ^b^ Clinical Laboratory Standards Institute (CLSI) guidelines [23].

**Table 3:** Distribution of 12 virulence-related genes detected by PCR in the six *Aeromonas lusitana* strains.

Strain	Source	*laf*	*act*	*ast*	*alt*	*aerA*	Lipase Genes	Serine Protease Genes	*ascF-G*	*ascV*	*aexT*	*stx1*	*stx2*
ESV-351	Gills of rainbow trout	-	+	-	-	+	+	+	-	-	-	-	-
CECT 7828^T^	Untreated water	+	-	-	-	+	+	+	+	+	+	-	-
A.136/15	Untreated water	+	-	-	-	+	+	+	+	+	-	-	-
L8-3	Vegetables	+	-	-	-	+	+	-	+	+	-	-	-
L10-4	Vegetables	+	+	-	-	+	+	+	-	-	-	-	-
A.2876	Untreated water	-	+	-	-	+	+	+	-	-	-	-	-

**Table 4:** Comparison of the main genomic characteristics of *Aeromonas lusitana* ESV-351 and CECT 7828^T^ strains, with the four closely related species and the six species that have closed genomes available at the NCBI database.

Species	Size (Mb)	Average	Protein Coding Genes	Average	tRNAs	Average	G+C (%)	Average
*A. lusitana* ESV-351 *A. lusitana* CECT 7828^T^	4.74 4.55	4.64	4251 4069	4160	110 98	104	60.7 61.0	60.8
**Closest species**								
*A. tecta* CECT 7028^T^ *A. eucrenophila* CECT 4224^T^ *A. aquatica* CECT 8025^T^ *A. encheleia* CECT 4342^T^	4.75 4.54 4.58 4.47	4.56	4267 4098 4091 4051	4094	96 101 82 94	95	60.1 61.2 61.2 62.0	61.2
**Closed genomes available at Genbank**								
*A. salmonicida* A449 *A. hydrophila* ATCC 7966^T^ *A. caviae* Ae398 *A. veronii* B565 *A. salmonicida* 01-B526 *A. dhakensis* AAK1	4.70 4.74 4.43 4.55 4.75 4.81	4.72	4388 4128 3690 4057 4179 4214	4153	110 128 ND 102 111 ND	110	58.5 61.5 61.4 58.7 58.5 61.8	60.0

## Data Availability

Not applicable.

## Supplementary Materials

The following supporting information can be downloaded at: https://www.mdpi.com/article/10.3390/pathogens11111299/s1, Table S1: Phenotypic characteristic of the *Aeromonas lusitana* Mexican strain (ESV-351) isolated from gills of rainbow trout obtained with conventional biochemical tests and with the MicroScan (W/A) identification system.
