# Supplementary material for: First Record of the Rare Species Aeromonas lusitana from Rainbow Trout (Oncorhynchus mykiss, Walbaum): Comparative Analysis with the Existing Strains
Source: Pathogens. 2022 Nov 5;11(11):1299. doi: 10.3390/pathogens11111299 (PMC9692447; doi:10.3390/pathogens11111299)
Supplement: Supplementary file 1 [file pathogens-11-01299-s001.zip › pathogens-1971542-supplementary.pdf]

Supplementary Table S1. Phenotypic characteristic of the *Aeromonas lusitana* Mexican strain (ESV-351) isolated from gills of rainbow trout obtained with conventional biochemical tests and with the MicroScan (W/A) identification system.

	Biochemical results			Biochemical results	
	Conventional	MicroScan		Conventional	MicroScan
**Test**			**Test**		
Motility	+	ND	Acid from:		
Oxidase	+	ND	D-adonitol	ND	-
Catalase	+	ND	D-cellobiose	-	ND
ADH	+	-	D-mannitol	+	ND
LDC	+	-	D-sorbitol	-	-
ODC	-	-	Glucose	ND	+
Tryptophan deaminase	ND	-	Glycerol	+	ND
Indole	+	-[a]	L- Arabinose	-	ND
ONPG	+	-	L- Rhamnose	-	ND
Urea	-	-	D-lactose	-	ND
H_2S	-	-	D-mellobiose	ND	-
VP	-	-	*m*-Inositol	-	-
MR	+	ND	D-raffinose	ND	ND
Gelatin	+	ND	D-sacarose	+	ND
DNase	+	ND	Salicin	+	ND
Glucose (gas)	+	ND	Sucrose	-	-
Nitrate reduction	+	+			
β–Hemolysis	+		Hydrolysis of:		
			Aesculin	+	-
Utilization of:			SDS	-	ND
Citrate	+	-	Starch	+	ND
DL-Lactate	-	ND			
Malonate	ND	-	Resistance to :		
Acetamide	ND	-	Chepalothin	ND	+
Tartrate	ND	-	Colistin	ND	-
			Kanamycin	ND	+
Growth in:			Nitrofurantoin	ND	-
0% NaCl	+	ND	O /129[b] (150 µg)	+	ND
4.5% NaCl	+	ND	Penicillin	ND	+
6% NaCl	-	ND	Tobramycin	ND	-
Cetridime	ND	-			
Growth at 42°C	-	ND			

ADH: Arginine dihydrolase; LDC: Lysine decarboxylase; ODC: Ornithine decarboxylase; ONPG: *o*-nitrophenyl-β-D-galactopyranoside; VP: Voges-Proskauer; MR: Methyl Red. [a] Negative in two of three repetitions; [b] Vibriostatic agent (2,4-diamino-6,7diisopropylpteridine) (150 µg / disk); ND: Not Done.
